# Supplementary material for: Complexome profiling of the Chlamydomonas psb28 mutant reveals TEF5 as an early PSII assembly factor
Source: Plant Cell. 2025 Mar 17;37(6):koaf055. doi: 10.1093/plcell/koaf055 (PMC12164590; doi:10.1093/plcell/koaf055)
Supplement: koaf055_Supplementary_Data [file koaf055_supplementary_data.zip › psb28_interactive.zip!/plots/intensity/qualityPlots/peptides/GGR1_Cre01.g050950.t1.2.html]

### Cre01.g050950.t1.2

Trivial name: GGR1  
SeqCoverage [median]: 59.50  
Mass: 55.61 kDa  
  
 

| Sequence | MissedCleavage | Mass [Da] | wtIntensity | muIntensity | Score |
| --- | --- | --- | --- | --- | --- |
| ANEERVMAA | 1 | 989.46 | 61718.20 | 37259.64 | 176.75 |
| AQKLGANIVNGLFMR | 1 | 1630.90 | 0.00 | 8101.41 | 52.82 |
| AVQSVFAVK | 0 | 947.54 | 2163.20 | 2626.58 | 87.32 |
| DGAALEGR | 0 | 787.38 | 9518.97 | 11654.30 | 145.34 |
| DGAALEGRK | 1 | 915.48 | 1015.31 | 0.00 | 89.36 |
| EIDAGEYDYAIAFQER | 0 | 1888.85 | 11681.07 | 40405.81 | 340.14 |
| EVFDDYLR | 0 | 1055.49 | 220433.82 | 337852.33 | 198.64 |
| GGVETFLLER | 0 | 1119.59 | 186907.10 | 328335.20 | 235.65 |
| GGVETFLLERK | 1 | 1247.69 | 0.00 | 2789.99 | 66.60 |
| GRVALVGDAAGYVTK | 1 | 1475.81 | 0.00 | 2014.84 | 97.80 |
| IAKEIDAGEYDYAIAFQER | 1 | 2201.06 | 11550.70 | 44633.20 | 137.89 |
| IIRVEAHPIPEHPRPR | 2 | 1916.09 | 2154.82 | 21922.88 | 90.75 |
| IRIPDDK | 1 | 855.48 | 14520.03 | 17670.79 | 180.18 |
| IRIPDDKMK | 2 | 1114.62 | 85623.49 | 112453.37 | 120.33 |
| LGANIVNGLFMR | 0 | 1303.71 | 7008.22 | 61117.23 | 235.65 |
| MAAEAIVEGSANGTK | 0 | 1447.70 | 0.00 | 1187.77 | 156.36 |
| MGKPATLEVDMIIGADGANSR | 1 | 2145.06 | 88008.77 | 248305.49 | 147.08 |
| MISPSNR | 0 | 803.40 | 103254.09 | 38479.96 | 130.04 |
| MISPSNREVDVGK | 1 | 1430.72 | 20720.23 | 14489.95 | 89.44 |
| MKMISPSNR | 1 | 1062.53 | 73764.15 | 62992.48 | 93.60 |
| MTFDSYLYK | 0 | 1166.53 | 149064.03 | 236933.69 | 217.02 |
| PATLEVDMIIGADGANSR | 0 | 1828.90 | 1574.46 | 1413.59 | 213.18 |
| QYQQATR | 0 | 893.44 | 171205.01 | 86094.20 | 217.21 |
| QYQQATRDR | 1 | 1164.56 | 1024.46 | 0.00 | 74.17 |
| REVFDDYLR | 1 | 1211.59 | 4338.58 | 29852.24 | 145.89 |
| SEQQSAEGPFTIHYNSYEDGSK | 0 | 2473.07 | 50595.92 | 181493.37 | 207.24 |
| SVNSKSVNVSFGSK | 1 | 1438.74 | 1012.05 | 0.00 | 32.73 |
| SVNVSFGSK | 0 | 923.47 | 221745.54 | 277577.18 | 199.89 |
| SVNVSFGSKANEER | 1 | 1522.74 | 4596.24 | 4295.98 | 89.63 |
| TAIKQYQQATR | 1 | 1306.70 | 44104.17 | 47987.49 | 194.39 |
| TEGGKIIR | 1 | 872.51 | 1719.65 | 0.00 | 89.55 |
| TVSSILR | 0 | 774.46 | 195299.16 | 103170.92 | 118.69 |
| TVVPGNPLDDVK | 0 | 1252.67 | 346842.42 | 453286.23 | 147.28 |
| TVVPGNPLDDVKLLVR | 1 | 1734.00 | 7416.54 | 50789.62 | 156.00 |
| VALVGDAAGYVTK | 0 | 1262.69 | 199080.63 | 410694.52 | 199.59 |
| VEAHPIPEHPR | 0 | 1280.66 | 0.00 | 605.34 | 66.60 |
| VEAHPIPEHPRPR | 1 | 1533.82 | 52888.83 | 71626.82 | 151.70 |
| VKTEGGKIIR | 2 | 1099.67 | 2708.47 | 671.91 | 85.96 |
| VLDILQK | 0 | 827.51 | 165987.67 | 152814.75 | 170.72 |
| VLDILQKVFYR | 1 | 1392.81 | 0.00 | 9813.06 | 107.24 |
| VYLDKWDR | 1 | 1093.56 | 7439.22 | 14379.23 | 123.72 |
| VYLDKWDRK | 2 | 1221.65 | 1492.09 | 1984.41 | 91.58 |
| YDHVAVGTGTVVNK | 0 | 1458.75 | 451824.09 | 381158.39 | 211.52 |
| YDHVAVGTGTVVNKTAIK | 1 | 1872.01 | 2854.27 | 4473.67 | 123.76 |
| YWTTYK | 0 | 860.41 | 5180.31 | 4558.74 | 103.28 |
|  |  |  | 2990035.9 | 3919968.6 |  |
